# Genomic Regions Associated with Multiple Sclerosis Are Active in B Cells

**DOI:** 10.1371/journal.pone.0032281

**Published:** 2012-03-02

**Authors:** Giulio Disanto, Geir Kjetil Sandve, Antonio J. Berlanga-Taylor, Julia M. Morahan, Ruth Dobson, Gavin Giovannoni, Sreeram V. Ramagopalan

**Affiliations:** 1 Wellcome Trust Centre for Human Genetics, University of Oxford, Oxford, United Kingdom; 2 Nuffield Department of Clinical Neurosciences (Clinical Neurology), University of Oxford, John Radcliffe Hospital, Oxford, United Kingdom; 3 Department of Informatics, University of Oslo, Blindern, Oslo, Norway; 4 Nuffield Department of Clinical Medicine, University of Oxford, John Radcliffe Hospital, Oxford, United Kingdom; 5 Blizard Institute, Queen Mary University of London, Barts and The London School of Medicine and Dentistry, London, United Kingdom; Julius-Maximilians-Universität Würzburg, Germany

## Abstract

More than 50 genomic regions have now been shown to influence the risk of multiple sclerosis (MS). However, the mechanisms of action, and the cell types in which these associated variants act at the molecular level remain largely unknown. This is especially true for associated regions containing no known genes. Given the evidence for a role for B cells in MS, we hypothesized that MS associated genomic regions co-localized with regions which are functionally active in B cells. We used publicly available data on 1) MS associated regions and single nucleotide polymorphisms (SNPs) and 2) chromatin profiling in B cells as well as three additional cell types thought to be unrelated to MS (hepatocytes, fibroblasts and keratinocytes). Genomic intervals and SNPs were tested for overlap using the Genomic Hyperbrowser. We found that MS associated regions are significantly enriched in strong enhancer, active promoter and strong transcribed regions (*p* = 0.00005) and that this overlap is significantly higher in B cells than control cells. In addition, MS associated SNPs also land in active promoter (*p* = 0.00005) and enhancer regions more than expected by chance (strong enhancer *p* = 0.0006; weak enhancer *p* = 0.00005). These results confirm the important role of the immune system and specifically B cells in MS and suggest that MS risk variants exert a gene regulatory role. Previous studies assessing MS risk variants in T cells may be missing important effects in B cells. Similar analyses in other immunological cell types relevant to MS and functional studies are necessary to fully elucidate how genes contribute to MS pathogenesis.

## Introduction

Multiple sclerosis (MS) is a complex immune mediated disorder of the central nervous system which arises from a combination of genetic and environmental factors and their interactions [1]. A recent genome wide association study (GWAS) involving more than nine thousand MS patients found evidence for association of MS with 57 genomic regions [2]. However, there remains limited understanding as to how these variants are involved in MS development.

Although T cells have traditionally been thought to mediate MS pathophysiology, attention to the role of B cells is increasing [3]–[4]. The success of Rituximab (an anti-CD20 monoclonal antibody) [5] heightened this interest, and a number of other anti-CD20 monoclonal antibodies are undergoing clinical trials [6].

The regulation of genes can be just as important as the proteins they encode. Regulatory elements in the genome are much harder to identify than protein-coding genes because they lack distinguishing sequence signatures. Moreover, many regulatory elements function only in certain cell types and conditions [7].

Chromatin profiling is a powerful means of genome annotation and detection of regulatory activity. The chromatin landscape of a cell is distinctive for a specific cell type and among other roles determines which regions of the genome are accessible to the binding of transcription factors and whether transcription occurs or is repressed. A recent study mapped a number of chromatin marks across nine cell types to systematically characterize regulatory elements and their cell-type specificities. These included enhancer elements (DNA sequences able to modulate gene expression through the binding of transcription factors to them), promoter regions (DNA regions located near the transcription start site of a gene which facilitate the binding of RNA polymerase and the initiation of transcription), polycomb repressed (DNA regions in which gene expression is actively repressed by the binding of polycomb group proteins), heterochromatin (large portions of DNA which are densely packed and therefore less accessible to transcription factors), insulator sites (DNA elements bound by the zinc finger protein CTCF which functions as an enhancer-blocking element), transcribed regions and finally repetitive/copy number variations (CNV, DNA regions characterized by a variable number of copies between individuals). Among the cell types profiled were B cells, hepatocytes, fibroblasts and keratinocytes [8].

A large proportion of SNPs associated with MS do not lie in the coding regions of genes and therefore are likely to influence disease risk through a gene regulatory role. It is plausible that genetic variants associated with a certain disease act through influencing the particular cell type(s) that trigger disease onset. Therefore, one would expect to observe an overlap between genomic regions associated with disease risk and those which are active in the causative cell type(s). The aim of this study was to assess whether genomic regions that have been associated with MS significantly overlap with active regulatory regions in B cells and whether this overlap is higher than that observed in non-immunological cell types. This potentially provides us with relevant information regarding the importance of the immune system and B cells as mediators of disease in MS.

## Methods

### Data acquisition

Genetic variants associated with MS risk were obtained from the recent GWAS performed by the International Multiple Sclerosis Genetics Consortium (IMSGC) and the Wellcome Trust Case Control Consortium 2 (WTCCC2) [2]. MS regions were defined as genomic intervals of 0.25 cM centred on the lead associated SNP. The chromatin profiles of B-lymphoblastoid cells (GM12878), hepatocellular carcinoma cells (HepG2), normal lung fibroblasts (NHLF) and normal epidermal keratinocytes (NHEK) were obtained from the ENCODE project [8]. Briefly, chromatin immunoprecipitation followed by massively parallel DNA sequencing (ChIP-seq) and expression data were used to identify different classes of chromatin states: active promoter (AP), weak promoter (WP), poised promoter (PP), strong enhancer (SE), weak enhancer (WE), polycomb repressed (PR), heterochromatic (H), insulator (I), strongly transcribed (ST), weakly transcribed (WT) and repetitive/CNV (Rep/CNV) [8].

### Overlap analysis

All analyses were performed using the Genomic Hyperbrowser (http://hyperbrowser.uio.no/hb/) [9]. The enrichment of MS regions in a certain chromatin state (e.g. AP) was calculated as the ratio of the proportion of AP intervals covered by MS regions, to the proportion of non-AP intervals covered by MS regions. In order to assess whether the overlap between MS regions and a certain chromatin state was higher than expected by chance, a permutation based analysis was performed. We defined a null model for which the location of individual chromatin intervals varied randomly, while preserving the empirical segment and inter-segment length distribution of chromatin intervals. MS regions were fixed. The number of overlapping base pairs between the two tracks was calculated for the real data, as well as for 20,000 Monte Carlo samples from the null model. The p-value was calculated in the usual way, i.e. as the proportion of Monte Carlo samples being equal to or more extreme than the observed overlap. These analyses were performed on both a global (whole genome) and local (chromosome arms) scale and for each cell type. For local analyses p-values were adjusted to a FDR of 10%.

When comparing B cells to non immunological cell types, case-control tracks were created for each chromatin state by removing all parts of chromatin intervals that overlapped between B and control cells and marking the remaining intervals as case (B cell specific intervals) and control (other cell type specific intervals). P-values were computed by a Monte Carlo procedure, in which the case-control labels of chromatin intervals were randomly permuted. The observed base pair overlap between case intervals and MS regions were compared against the corresponding distribution for 20,000 Monte Carlo samples in the usual way. The fold enrichment difference in overlap between B and control cells was calculated as the ratio between the proportions of case and control intervals that overlapped with MS regions.

Finally we tested whether MS associated SNPs (primary SNPs) and SNPs in perfect linkage disequilibrium (LD) with primary SNPs (r^2^ = 1) were located within certain chromatin states more than expected by chance as described above for MS regions.

## Results

### Active chromatin states in B cells overlap with MS regions

Our first aim was to assess whether and where in the genome a particular chromatin state in B cells significantly overlapped with MS regions. The enrichment of MS regions in different chromatin states and the significance of the overlap are presented in Table 1. On a global scale (whole genome) enrichment values varied considerably between different chromatin states ranging from 0.34 in H to 3.07 in SE regions. When testing statistical significance, MS regions overlapped with promoter (AP and WP *p* = 0.00005; PP *p* = 0.0005), enhancer (*p* = 0.00005) and transcribed (*p* = 0.00005) regions more than expected by chance.

**Table 1:** Enrichment of MS regions in chromatin states on both a global (whole genome) and local (chromosome arms) scale in B cells.

BINS	Active promoter	Weak promoter	Poised promoter	Strong enhancer	Weak enhancer	Polycomb repressed	Hetero-chromatic	Insulator	Strong transcribed	Weak transcribed	Rep/CNV	MS SNPs and putative associated genes
Whole genome	2.72 *	2.13 *	2.35 *	3.07 *	2.22 *	1.35	0.34	1.2	2.57 *	1.92 *	1.013	
chr1p	1.28	1.20	0.41	2.12 *	1.48 *	0.45	0.69	0.96	1.10	1.47	4.39 *	rs4648356 (*MMEL1/TNFRSF14*); rs11810217 (*EVI5*); rs11581062 (*VCAM1*); rs1335532 (*CD58*)
chr1q	2.04*	0.46	0.00	4.65 *	3.12 *	2.78	0.33	1.72	0.90	1.57	0.00	rs1323292 (*RGS1*); rs7522462 (*C1orf106/KIF21B*)
chr2p	3.22 *	2.73 *	4.12	12.78 *	3.35 *	1.47	0.31	1.72	0.44	1.12	0.00	rs12466022 (no gene); rs7595037 (*PLEK*)
chr2q	3.18 *	3.26 *	1.89	1.45 *	1.89 *	2.28	0.32	1.23	3.16 *	1.69	0.50	rs17174870 (*MERTK*); rs882300 (*CXCR4*); rs10201872 (*SP140*)
chr3p	0.00	0.00	20.01 *	0.00	0.28	3.18	2.33	0.15	0.00	0.12	0.54	rs11129295 (*EOMES*); rs669607 (no gene)
chr3q	3.11 *	1.99 *	1.05	7.35 *	2.27 *	0.22	0.14	0.87	5.88 *	2.24 *	0.65	rs2028597 (*CBLB*); rs2293370 (*TMEM39A/CD80*); rs9282641 (*CD86*); rs2243123 (*IL12A*)
chr4q	6.20 *	4.16 *	0.00	4.16 *	1.40 *	0.02	0.15	0.70	9.65 *	3.66 *	0.94	rs228614 (*NFKB1*)
chr5p	0.61	0.91	4.10	1.71 *	3.55 *	3.41 *	0.59	2.38 *	0.23	1.79	0.00	rs6897932 (*IL7R*); rs4613763 (*PTGER4*)
chr5q	5.85 *	4.21 *	1.17	1.42 *	1.51 *	0.82	0.48	1.22	2.74	1.06	0.35	rs2546890 (*IL12B*)
chr6q	0.51	0.82	2.71 *	3.21 *	2.28 *	4.03 *	0.59	1.49	0.04	1.12	0.55	rs12212193 (*BACH2*); rs802734 (*THEMIS*); rs11154801 (*MYB/AHI1*); rs17066096 (*IL22RA2*); rs13192841 (no gene); rs1738074 (*TAGAP*)
chr7q	2.87 *	1.67	0.00	1.65 *	0.91	2.52	0.22	0.95	6.34 *	1.71	0.00	rs354033 (*ZNF746*)
chr8q	0.87	1.04	0.00	0.32	1.60 *	0.42	0.66	0.61	2.53	1.47	0.13	rs1520333 (*IL7*); rs4410871 (*MYC*); rs2019960 (*PVT1*)
chr10p	4.40 *	7.65 *	4.42	4.98 *	7.49 *	2.93	0.13	1.35	1.98	1.85	0.51	rs3118470 (*IL2RA*)
chr10q	3.03 *	2.46 *	7.89 *	3.02 *	2.34 *	1.90	0.11	0.89	8.78 *	1.64	0.66	rs1250550 (*ZMIZ1*); rs7923837 (*HHEX*)
chr11q	2.55 *	2.98 *	8.53 *	1.93 *	3.01 *	1.34	0.17	0.93	3.62 *	2.63 *	0.21	rs650258 (*CD6*); rs630923 (*CXCR5*)
chr12p	4.55 *	1.94 *	0.00	3.97 *	2.31 *	0.66	0.34	1.34	2.03	1.79	0.67	rs1800693 (*TNFRSF1A*); rs10466829 (*CLECL1*)
chr12q	3.66 *	2.52 *	3.68 *	1.66 *	1.73 *	0.73	0.26	1.09	3.20 *	2.35 *	0.64	rs12368653 (*CYP27B1*); rs949143 (*ARL6IP4*)
chr14q	2.56 *	1.37	0.00	3.42 *	2.24 *	0.23	0.60	1.25	0.20	1.93 *	1.08	rs4902647 (*ZFP36L1*); rs2300603 (*BATF*); rs2119704 (*GALC/GPR65*)
chr16p	1.33	0.35	0.00	6.65 *	3.54 *	0.00	0.14	0.66	1.63	2.64 *	0.34	rs2744148 (*SOX8*); rs7200786 (*CLEC16A/CIITA*)
chr16q	0.78	1.27	0.00	16.42 *	8.29 *	0.00	0.10	1.65	0.00	3.07	3.47	rs13333054 (*IRF8*)
chr17q	2.48 *	2.07 *	0.48	2.38 *	1.77 *	0.37	0.21	0.74	2.66 *	1.81 *	1.02	rs9891119 (*STAT3*); rs180515 (*RPS6KB1*)
chr18q	7.70 *	5.81 *	4.78	3.77 *	3.58 *	2.16	0.08	1.06	11.92 *	2.36	0.00	rs7238078 (*MALT1*);
chr19p	1.66 *	1.87 *	4.79 *	2.54 *	2.33 *	2.13	0.19	1.90 *	1.54	1.66 *	0.29	rs1077667 (*TNFSF14*); rs8112449 (*TYK2/ICAM3*); rs874628 (*MPV17L2/IL12RB1*)
chr19q	0.25	0.62	2.82	2.17 *	1.51 *	2.26	1.78	1.62	0.00	0.43	0.14	rs2303759 (*DKKL1/CD37*)
chr20q	3.12 *	2.53 *	5.08 *	1.37	1.92 *	2.52 *	0.31	1.72	1.30	2.00 *	0.18	rs2425752 (*CD40*); rs2248359 (*CYP24A1*); rs6062314 (*TNFRSF6B*)
chr22q	3.15 *	2.79 *	0.31	1.50 *	1.26 *	0.33	0.32	1.01	2.48	1.66	1.09	rs2283792 (*MAPK1*); rs140522 (*SCO2*)

Bold and * indicate the presence of a statistically significant overlap.

In order to assess whether the significant global overlap was homogeneously distributed across the genome or resulted from particularly highly enriched regions, the same analysis was performed on a local scale by dividing the whole genome into chromosome arms. This resulted in 43 different ‘bins’ of which 17 had to be excluded due to the absence of MS associated regions in those chromosome arms leaving a total of 26 bins. Out of these 26 bins, statistically significant overlap was found in 18 for AP, 15 for WP, 7 for PP, 23 for SE, 24 for WE, 3 for PR, 0 for H, 2 for I, 9 for ST, 9 for WT and 1 for Rep/CNV chromatin states (Table 1). As expected, the chromatin states with significant overlap on a global scale were those with the highest number of significant bins. SE and WE regions showed the most homogeneously distributed overlap, being significant in all but 3 and 2 bins respectively. The overlap of promoter and transcribed regions appeared more dependent on particular bins.

### Active chromatin states in B cells overlap with MS regions more than in non immunological cell types

On its own, the presence of an overlap between MS regions and active chromatin states in B cells is not sufficient to indicate that B cells are relevant to MS pathogenesis. Both MS regions and active chromatin states could just be more likely to be near commonly transcribed genes, giving rise to co-localization in the absence of any direct relationship between the MS-associated regions and chromatin states. To rule out this hypothesis we tested 3 additional cell types (hepatocytes, fibroblasts and keratinocytes) that, based on current knowledge, are not implicated in MS pathogenesis. Enrichment values, global significance and number of significant bins are presented in Table 2.

**Table 2:** Enrichment, global significance and number of significant bins in B and control cells.

CHROMATIN STATE	MEASURE	B CELLS	HEPATOCYTES	FIBROBLASTS	KERATINOCYTES
	Global significance	**YES (** ***p*** ** = 0.00005)**	**YES (** ***p*** ** = 0.00005)**	**YES (** ***p*** ** = 0.00005)**	**YES (** ***p*** ** = 0.00015)**
Active promoter	Significant bins	**18**	**9**	**11**	**6**
	Global enrichment	**2.721**	**2.114**	**2.207**	**1.95**
	Global significance	**YES (** ***p*** ** = 0.00005)**	**YES (** ***p*** ** = 0.00005)**	**YES (** ***p*** ** = 0.0001)**	**YES (** ***p*** ** = 0.00005)**
Weak promoter	Significant bins	**15**	**11**	**7**	**10**
	Global enrichment	**2.134**	**2.121**	**1.803**	**1.949**
	Global significance	**YES (** ***p*** ** = 0.0005)**	Maybe (*p* = 0.01975)	**YES (** ***p*** ** = 0.0022)**	**YES (** ***p*** ** = 0.0013)**
Poised promoter	Significant bins	**7**	5	**4**	**4**
	Global enrichment	**2.352**	1.935	**2.057**	**2.011**
	Global significance	**YES (** ***p*** ** = 0.00005)**	**YES (** ***p*** ** = 0.0016)**	**YES (** ***p*** ** = 0.00005)**	**YES (** ***p*** ** = 0.00065)**
Strong enhancer	Significant bins	**23**	**1**	**6**	**7**
	Global enrichment	**3.074**	**1.908**	**1.886**	**1.577**
	Global significance	**YES (** ***p*** ** = 0.00005)**	**YES (** ***p*** ** = 0.00005)**	**YES (** ***p*** ** = 0.00035)**	**YES (** ***p*** ** = 0.00005)**
Weak enhancer	Significant bins	**24**	**12**	**4**	**10**
	Global enrichment	**2.222**	**1.747**	**1.436**	**1.528**
	Global significance	Maybe (*p* = 0.07035)	NO (*p* = 0.6002)	**YES (** ***p*** ** = 0.0056)**	NO (p = 0.1098)
Polycomb repressed	Significant bins	3	1	**8**	5
	Global enrichment	1.355	1.161	**1.574**	1.314
	Global significance	NO (*p* = 1)	NO (*p* = 1)	NO (*p* = 1)	NO (*p* = 1)
Heterochromatic	Significant bins	0	0	0	0
	Global enrichment	0.3363	0.4589	0.4273	0.4829
	Global significance	Maybe (*p* = 0.08100)	Maybe (*p* = 0.02090)	**YES (** ***p*** ** = 0.0015)**	**YES (** ***p*** ** = 0.00025)**
Insulator	Significant bins	2	5	**4**	**7**
	Global enrichment	1.205	1.267	**1.348**	**1.376**
	Global significance	**YES (** ***p*** ** = 0.00005)**	**YES (** ***p*** ** = 0.0004)**	**YES (** ***p*** ** = 0.0007)**	**YES (** ***p*** ** = 0.005)**
Strong transcribed	Significant bins	**9**	**7**	**8**	**7**
	Global enrichment	**2.575**	**2.171**	**2.133**	**1.868**
	Global significance	**YES (** ***p*** ** = 0.00005)**	**YES (** ***p*** ** = 0.0008)**	**YES (** ***p*** ** = 0.00005)**	**YES (** ***p*** ** = 0.00005)**
Weak transcribed	Significant bins	**9**	**3**	**8**	**7**
	Global enrichment	**1.919**	**1.668**	**1.836**	**1.811**
	Global significance	NO (*p* = 0.3547)	Maybe (*p* = 0.02565)	NO (*p* = 0.5321)	NO (*p* = 0.1582)
Repetitive/CNV	Significant bins	1	1	1	1
	Global enrichment	1.013	1.965	0.8118	1.351

Similarly to findings observed in B cells, promoter, enhancer and transcribed regions overlapped with MS regions more than expected by chance in all control cell types. However, the number of significant bins as well as the enrichment values tended to be higher in B cells than in other cell types. We explored this further by directly comparing the overlap in B cells with that of the control cells (Table 3). Strikingly the overlap between MS regions and AP, SE, WE and ST regions was significantly higher in B cells than in any of the other cell types. The highest fold enrichment differences were observed for higher activity states (AP, SE and ST).

**Table 3:** Comparison of overlaps between B cells and other cell types.

Chromatin state	Is overlap in B cells>Hepatocytes?		Is overlap in B cells>Fibroblasts?		Is overlap in B cells>Keratinocytes?	
	Significance	Fold difference	Significance	Fold difference	Significance	Fold difference
Active promoter	**YES (** ***p*** ** = 0.0001)**	**1.958**	**YES (** ***p*** ** = 0.0006)**	**1.888**	**YES (** ***p*** ** = 0.00005)**	**2.747**
Weak promoter	NO (*p* = 0.5972)	1.022	**YES (** ***p*** ** = 0.00075)**	**1.239**	**YES (** ***p*** ** = 0.0025)**	**1.068**
Poised promoter	**YES (** ***p*** ** = 0.00945)**	**1.259**	Maybe (*p* = 0.07815)	1.208	NO (*p* = 0.2274)	1.255
Strong enhancer	**YES (** ***p*** ** = 0.00005)**	**1.709**	**YES (** ***p*** ** = 0.00005)**	**1.772**	**YES (** ***p*** ** = 0.00005)**	**2.196**
Weak enhancer	**YES (** ***p*** ** = 0.00005)**	**1.306**	**YES (** ***p*** ** = 0.00005)**	**1.626**	**YES (** ***p*** ** = 0.00005)**	**1.513**
Polycomb repressed	NO (*p* = 0.1456)	1.213	NO (*p* = 1)	0.8860	NO (*p* = 0.9866)	1.119
Heterochromatic	NO (*p* = 1)	0.5286	NO (*p* = 1)	0.5938	NO (*p* = 1)	0.4705
Insulator	NO (*p* = 0.7215)	0.8227	NO (*p* = 0.3529)	0.9084	NO (*p* = 0.9425)	0.8002
Strong transcribed	**YES (** ***p*** ** = 0.0015)**	**1.486**	**YES (** ***p*** ** = 0.00005)**	**1.417**	**YES (** ***p*** ** = 0.00005)**	**2.027**
Weak transcribed	Maybe (p = 0.07585)	1.230	NO (*p* = 0.1152)	1.096	NO (*p* = 0.1358)	1.132
Repetitive/CNV	NO (*p* = 1)	0.1713	NO (*p* = 0.2893)	2.492	NO (*p* = 0.9999)	0.4362

### MS associated SNPs preferentially land in active promoter and enhancer regions

The presence of significant overlap between MS regions and certain active chromatin states in B cells supports an important role for the immune system in MS but does not provide any insight into how MS risk variants may be acting. We attempted to answer this question by looking at where in the genome MS associated SNPs, and SNPs in perfect LD (r^2^ = 1), preferentially land. A total of 452 SNPs were tested and enrichment of chromatin states for MS SNPs and significance of overlap are presented in Table 4. MS SNPs were located within AP, SE and WE intervals more than expected by chance. Weak evidence for overlap was also observed for WP, ST and WT regions.

**Table 4:** Enrichment and significance of the overlap between MS SNPs and chromatin states in B cells.

Chromatin state	MS SNPs		Significance	Enrichment
	Number	Percentage		
Active promoter	30	6.64	**YES (p = 0.00005)**	**9.202**
Weak promoter	9	1.99	Maybe (p = 0.0413)	2.907
Poised promoter	2	0.44	NO (p = 0.181)	2.72
Strong enhancer	35	7.74	**YES (p = 0.0006)**	**4.878**
Weak enhancer	57	12.61	**YES (p = 0.00005)**	**4.580**
Polycomb repressed	9	1.99	NO (p = 0.7278)	0.63
Heterochromatic	162	35.84	NO (p = 1)	0.216
Insulator	4	0.88	NO (p = 0.2574)	1.634
Strong transcribed	59	13.05	Maybe (p = 0.0529)	2.271
Weak transcribed	85	18.81	Maybe (p = 0.0357)	1.973
Repetitive/CNV	0	0.00	NO (p = 1)	0

**Figure 1:**
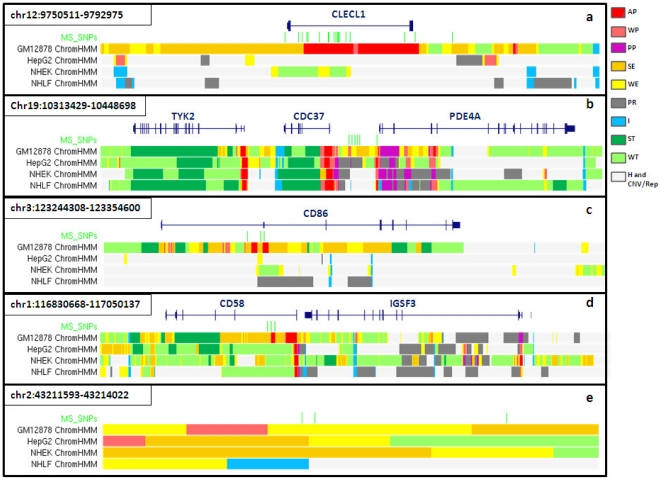
MS SNPs land in B cell specific promoter and enhancer elements in the regions of *CLECL1*, *TYK2*, *CD86*, and *CD58* (a–d); MS SNPs in a region with no candidate gene land in enhancer intervals (e). Chromatin states: AP = active promoter; WP = weak promoter; PP = poised promoter; SE = strong enhancer; WE = weak enhancer; PR = polycomb repressed; H = heterochromatic; I = insulator; ST = strong transcribed; WT = weak transcribed; CNV/Rep = CNV/repetitive. Cell lines: GM12878 = B cells; HepG2 = hepatocytes; NHEK = keratinocytes; NHLF = fibroblasts.

When examined in the light of the chromatin data, several regions seemed particularly interesting. For example MS associated SNPs in the regions of *CLECL1*, *CD86*, *TYK2* and *CD58* land in promoter and enhancer regions which are present in B cells but not in other cell types (Figure 1). We also looked at the position of MS SNPs in regions in which no candidate genes have been identified. Interestingly, rs12466022 on chromosome 2 and respective SNPs in LD landed in WE and SE intervals, while rs13192841 on chromosome 6 and respective SNPs in LD were located in WE and WT regions. The complete list of SNPs and overlapping chromatin states is available in supplementary material online (Table S1).

## Discussion

MS is a complex disorder of unknown aetiology. Here we show that genomic regions associated with MS overlap with AP, SE, WE and ST regions in B cells and that this occurs more than would be expected by chance, and more than was observed in 3 other cell types unrelated to MS pathogenesis. Notably, the overlap was particularly striking in SE and WE regions for which significance was reached in 23 and 24 out of the 26 analyzed bins respectively. This is in accordance with the previous observations that tissue-specific genes appear more dependent on enhancer than promoter elements [8]. Furthermore, we provide evidence that the associated SNPs preferentially land in promoter, enhancer and to a lesser extent transcribed regions. These findings have several important implications.

Firstly, this work further supports the immunological aetiology of MS [10]. Our findings are in agreement with those of a gene-ontology analysis of the genes located within MS associated regions, which showed a substantial overrepresentation of immune-related processes [2]. As compared to this type of analysis, our approach has the relative advantage of being independent of what is currently known on genes and cell types.

Secondly our observations provide further support for an important role for B cells in the pathogenesis of MS. The presence of oligoclonal bands in the cerebrospinal fluid is the most consistent immunological finding in MS, and this indicates abnormal B cell activation within the CNS of MS patients [11]. Furthermore B cell abnormalities influence both conversion to clinically definite MS, MRI activity, onset of relapses and disease progression [12]–[17]. Possibly the strongest evidence for B cells in MS comes from clinical trials showing that MRI activity and onset of relapses are significantly decreased after depletion of CD20+ B cells [5]. However, we must consider that certain chromatin features may be shared between B and other immune cell types, in particular T cells. Unfortunately a similarly detailed chromatin profile of T cells is not yet available and therefore a direct comparison between B and T cells could not be performed. Even if similar chromatin profiles exist between T and B cells, the attempts to understand the effects of genetic risk variants on T cell function [18] may be missing important effects in B cells. Given the increasing evidence for B-T cell interactions in MS [19]–[21], this analysis has the potential to greatly help the dissection of the roles played by these two cell types.

When MS SNPs rather than MS regions were analyzed, we found that MS SNPs were significantly more likely to land in AP, SE and WE regions than expected by chance perhaps suggesting that many of the associated SNPs may influence the risk of MS by modifying the binding of transcription factors and transcription in general. This is in agreement with previous observations [8]. For MS associated SNPs landing in non-genic regions, for the first time we are able to show a likely functional role in gene regulation. However these findings should be interpreted with caution for two reasons. First, the observed overlap between MS SNPs and active chromatin states may be consequent to the fact that MS SNPs land in MS regions, themselves enriched for active chromatin states. Secondly, a conclusive answer to this question can only come from functional studies which should investigate if and how MS variants affect the chromatin landscape and gene expression.

To conclude, genomic regions associated to MS susceptibility are active in B cells and causative SNPs may act by changing the chromatin landscape. Further similar analyses in other immunological cell types relevant to MS and functional studies are required to fully understand in which cells, at which stage and how MS genetic variants are acting.

## Supporting Information

Table S1 List of MS associated SNPs and SNPs in perfect LD (r^2^ = 1) with overlapping chromatin states.
(XLSX)
